# Basking in the sun: how mosses photosynthesise and survive in Antarctica

**DOI:** 10.1007/s11120-023-01040-y

**Published:** 2023-07-29

**Authors:** Hao Yin, Alicia V. Perera-Castro, Krystal L. Randall, Johanna D. Turnbull, Melinda J. Waterman, Jodie Dunn, Sharon A. Robinson

**Affiliations:** 1 Securing Antarctica’s Environmental Future, University of Wollongong, Wollongong, NSW 2522 Australia; 2 Centre for Sustainable Ecosystem Solutions, School of Earth, Atmospheric and Life Sciences, University of Wollongong, Wollongong, NSW 2522 Australia; 3 Universidad de La Laguna, La Laguna, Canary Islands Spain

**Keywords:** Antarctica, β-carotene, Climate change, Photosynthesis, Microclimate, Moss, Nutrients, Ozone depletion, Temperature, Ultraviolet-B radiation, UV-B, Water, Zeaxanthin

## Abstract

The Antarctic environment is extremely cold, windy and dry. Ozone depletion has resulted in increasing ultraviolet-B radiation, and increasing greenhouse gases and decreasing stratospheric ozone have altered Antarctica’s climate. How do mosses thrive photosynthetically in this harsh environment? Antarctic mosses take advantage of microclimates where the combination of protection from wind, sufficient melt water, nutrients from seabirds and optimal sunlight provides both photosynthetic energy and sufficient warmth for efficient metabolism. The amount of sunlight presents a challenge: more light creates warmer canopies which are optimal for photosynthetic enzymes but can contain excess light energy that could damage the photochemical apparatus. Antarctic mosses thus exhibit strong photoprotective potential in the form of xanthophyll cycle pigments. Conversion to zeaxanthin is high when conditions are most extreme, especially when water content is low. Antarctic mosses also produce UV screening compounds which are maintained in cell walls in some species and appear to protect from DNA damage under elevated UV-B radiation. These plants thus survive in one of the harshest places on Earth by taking advantage of the best real estate to optimise their metabolism. But survival is precarious and it remains to be seen if these strategies will still work as the Antarctic climate changes.

**Supplementary Information:**

The online version contains supplementary material available at 10.1007/s11120-023-01040-y.

## Introduction

**Fig. 1:**
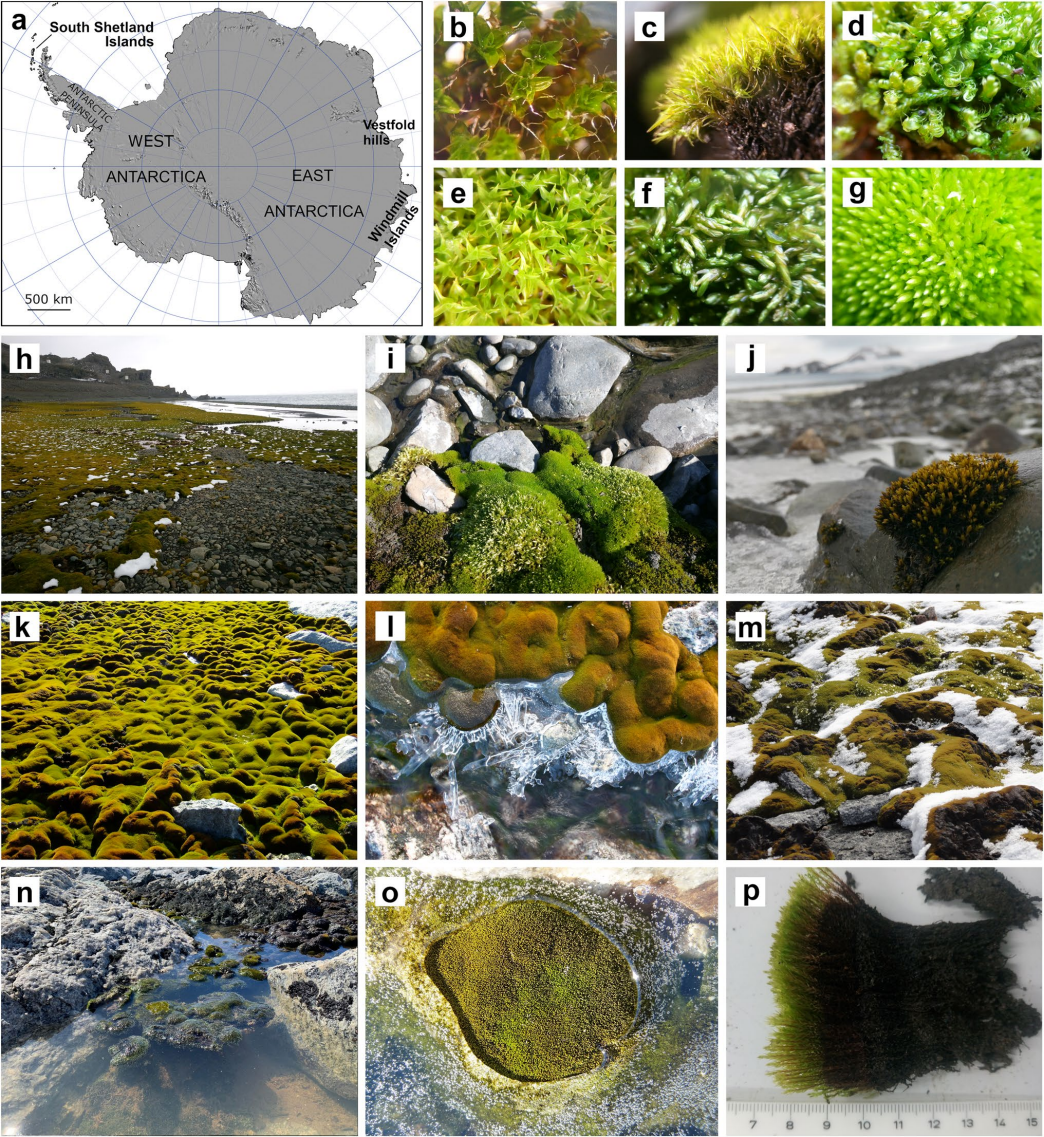
Antarctic map (**a**) and photographs of some of its mosses growing along streams in the South Shetland Islands (**b**–**j**, **p**) and Windmill Islands region (**k**–**o**): *Syntrichia magellanica* (**b**) *Chorisodontium aciphyllum* (**c**) *Sanionia georgicouncinata* **(d)** *Syntrichia filaris* **(e)** *Warnstorfia sarmentosa* **(f)** and *Bryum pseudotriquetrum* (**g**) turf of mosses on the shore of Livingston Island **(h)** and detail** (i)**. *Schistidium rivulare* growing as a small button on rock **(j)** Predominantly *Schistidium antarctici* in an extensive moss turf in Antarctic Specially Protected Area (ASPA) 135 **(k)** beside a frozen stream **(l)** covered by snow **(m)** or floating in liquid water after being displaced from turf by flooding **(n)** *B. pseudotriquetrum* with photosynthetically-derived bubbles trapped in the surrounding ice **(o)** Cross section of decades old *B. pseudotriquetrum* cushion with distinguishable fresh growth **(p)** Original source of map: the Scientific Committee on Antarctic Research modified with permission. Photographs Alicia Perera, Sharon Robinson, Jessica Bramley-Alves and Krystal Randall)

Antarctica is cold, dry and very windy. Its terrestrial flora is dominated by bryophytes (mosses and liverworts) and lichens. These are found all around the continent on coastal ice-free regions and even inland on nunataks (Fig. 1). In the maritime/peninsula region there are also two angiosperms: a cushion plant *Colobanthus quitensis* and the Antarctic hair grass *Deschampsia antarctica* (Cannone et al. 2016, 2022; Loisel et al. 2017; Torres-Mellado et al. 2011).

Some of the most extensive moss beds in East Antarctica are found in the Windmill Islands region near the Australian Antarctic Casey Station (66.2821° S, 110.5285° E; Fig. 1a, k–o). The proximity of these extensive moss beds to the station has enabled physiological studies to be performed over many decades with more than 35 papers published since the 1980s (including Lewis Smith 1999; Melick et al. 1994;; Melick and Seppelt 1994; Roser et al. 1992; Turnbull and Robinson 2009; Ashcroft et al. 2017; Nydahl et al. 2015; Bramley-Alves et al. 2014a, b; Lucieer et al. 2014; Bramley‐Alves et al. 2015; Hennion et al. 2006; Wasley et al. 2012; Robinson et al. 2000, see extra papers cited below) allowing us to better understand how these plants survive and thrive in this extreme environment.

Like any vegetation, mosses need water, sunlight and nutrients in order to photosynthesise and grow. Here we outline how these Antarctic mosses interact with their environment and our understanding of how they are able to photosynthesise under such harsh conditions. Light is vital for Antarctic mosses in multiple ways—not just as energy for photosynthesis. Infrared (IR) wavelengths provide the heat required to melt ice and the liquid water required for photochemical reactions (see Water from snow and ice). Photosynthetically active radiation (PAR) provides the energy for photosynthesis but IR energy also heats the moss beds to temperatures where photosynthetic processes are most effective (see Optimum temperature and Microclimate sections). When PAR energy exceeds the amount needed for photosynthesis it must be dissipated safely to avoid damage (see Protection section), and accompanying ultraviolet radiation can either signal a range of cellular processes (protective) or be damaging (Ultraviolet Radiation section). To produce the proteins that drive photosynthesis and the chlorophyll to fix sunlight, mosses need nutrients which are mainly provided from marine sources in Antarctica (Fertiliser section). Finally, we also discuss how the Antarctic environment is responding to climate change and ozone depletion and what this might mean in the future for its unique bryophyte flora (Future section).

### Water from snow and ice

In this cold desert, water is a key limiting factor (Davey and Rothery 1997; Convey et al. 2018; Robinson et al 2003; Colesie et al 2022). In East Antarctica water can come from snow (precipitated or blown) and melt water flows. In order to cope with the extreme environment, the bryophytes and lichens, along with the invertebrates that live within them, must be able to withstand frequent cycles of desiccation and rehydration, and freezing and thawing. Due to their poikilohydric nature, mosses tolerate up to nine months a year dried and frozen under snow and then manage to grow during the short summer season when ice melts and freshwater is available. In winter the snow cover acts to buffer them from the worst of the extreme winter temperatures, below − 40 °C on the coast and even colder inland. Whilst they are under snow mosses are also protected from wind damage. Mosses are found wherever they can obtain water. Some mosses grow in or around the edges of meltlakes but most are fed by ephemeral streams with lush turfs occurring where meltwater flows throughout the summer (Fig. 1h, k).

**Fig. 2:**
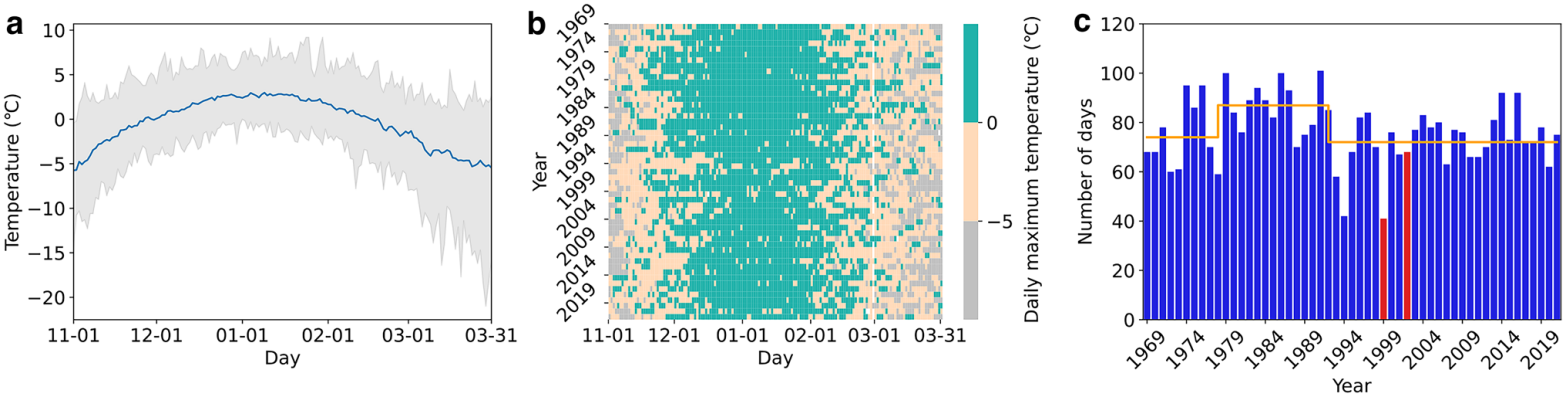
Temperature data collected at Casey Station, Antarctica by the Bureau of Meteorology (BOM) from 1969 to 2022 summarised here for 1^st^ November to 31^st^ March. **(a)** Daily temperature range is shown as grey shading with mean temperature shown as blue line. Free water is available to sustain plants once snow starts to melt in spring. **(b)** Daily maximum air temperature where green pixels represent the number of days in the spring/summer growing season when the maximum daily air temperature (24 h after 9 am) is above 0 °C, snow is likely to melt and mosses are fully exposed. At maximum daily temperatures between 0 and –5 °C some moss metabolism may occur on sunny days (beige pixels). Grey pixels represent days when air temperatures remained below –5 °C and mosses were likely dormant. **(c)** Number of days over the growing season when maximum temperature exceeded 0 °C. The red columns indicate the two seasons referred to in the text (1999–2000 and 2002–2003). The orange line represents mean values before and after identified changepoints in 1979 and 1993. (N.B. BOM moved from Casey tunnel location to the current site in 1989, see Robinson et al. 2018)

Temperature is an especially strong driver of plant growth in Antarctica as it determines availability of free water as well as directly affecting metabolism (Fig. 2a). Although maximum temperatures can exceed 0 °C at coastal locations like Casey throughout the year, they are most common in December and January; the only months when mean maximum temperatures above 0 °C occur (Fig. 2a).

Free water becomes available as snow starts to melt in spring. Once air temperature is above 0 °C (Fig. 2a, b), snow is likely to have melted and mosses will be fully exposed. When air temperatures are between 0 and –5 °C some metabolism may occur on sunny days, either under snow or if snow has already melted and moss is exposed (Melick and Seppelt 1992). This is probably more likely in late spring and summer and less likely in autumn once snow banks have melted and retreated and mosses are desiccated. Once temperatures drop below –5 °C mosses are likely to be frozen, dry and dormant (Cannone et al 2017).

Defined by this melt water availability, the summer growing season is therefore very short, ranging between 40 and 100 days of melt per season at Casey since 1969 (Fig. 2c). The number of melt days has shown a decline since 1993 with summers averaging 87 days of potential melt in the 1980s compared with 72 since 2000. The effects of cooler summer temperatures, increased wind speeds and less melt is also apparent from moss cores collected from the region which show evidence of a drying trend (Robinson et al. 2018). Modern carbon dating and studies of changes in stable isotopes of carbon (δ^13^; Clarke et al. 2012) down intact moss shoots have revealed a drying trend in the Windmill Islands for the 50 years up to 2012 (Robinson et al. 2018). This corresponds with a period of cooler temperatures and drying due to increased wind speeds, factors connected to the climatic effects of ozone depletion described below.

**Fig. 3:**
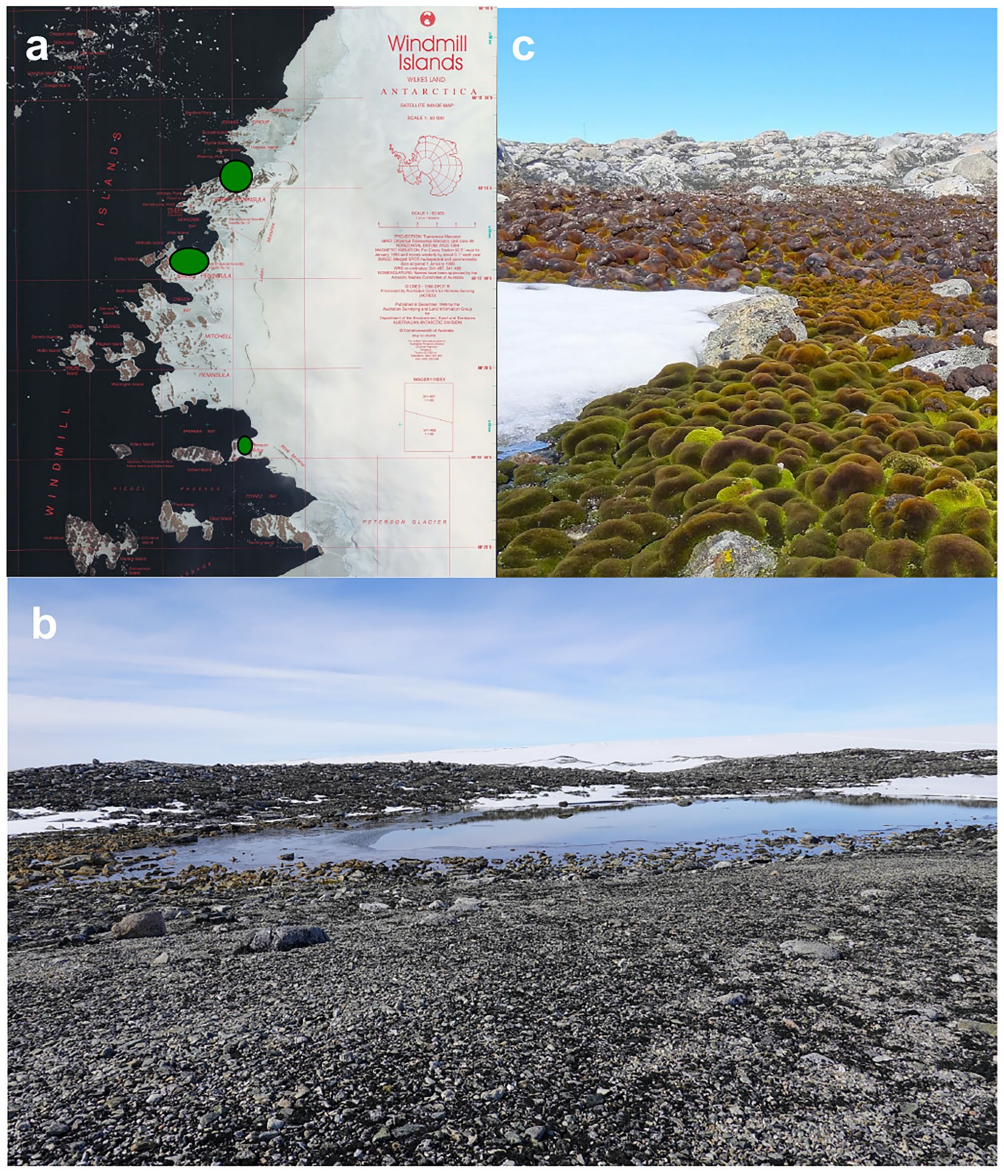
Sites with rich nutrient supply from ancient penguin colonies support extensive moss beds. Windmill Islands satellite image showing locations of extensive moss beds (green ovals) (**a)**. Photograph of ASPA135, Bailey Peninsula showing a ridge (foreground) covered in Adélie beak sized pebbles deposited by nesting penguins 3000–8000 years ago (**b)**. In depressions below these ancient nest sites, mosses thrive fed by melt water from nearby snowbanks, as shown in (**c**) with moss below snowbank, green and healthy, but above the snowline, only lichens and dry moss are found (grey or red coloured moss indicating moribund and very stressed moss, respectively). (Photographs Sharon Robinson, AAD map 95/056 https://data.aad.gov.au/aadc/mapcat/display_map.cfm?map_id=10)

Water can be deposited directly onto vegetation as precipitation (Fig. 1m) but mainly drains from nearby glaciers and smaller snow banks which melt in spring (Fig. 1h, n; Fig. 3c). Ephemeral streams deliver unreliable water with the best moss beds found where multiple streams ensure a steady supply of water for the whole summer (Fig. 1h; k, n, Fig. 3c).

### Optimum temperatures for net CO_2_ assimilation

Growth rate of mosses around the world is related to the balance between two processes: carbon uptake through photosynthesis and carbon loss through respiration. Therefore, the carbon budget can be maximised  through optimal daytime photosynthesis and suppressed respiration during night. Since high temperatures can enhance both photosynthesis and respiration rates, especially under well-watered conditions (Wilson 1990), the daytime response to temperature must be evaluated as the light-saturated net CO_2_ assimilation, considering both components of carbon dynamics. Some Antarctic mosses present maximum values of electron transport rate (ETR) and diurnal net CO_2_ assimilation at canopy temperatures higher than 20 °C (Perera-Castro et al. 2020 and references therein), with extremes of 25–30 °C reported for *Bryum pseudotriquetrum* and *Ceratodon purpureus*. In some species, such as *Schistidium antarctici*, high discrepancies between studies have been reported with optimum temperatures ranging from 0 to 30 °C (Kappen et al. 1989; Davey and Rothery 1997; Block et al. 2009; Wilson 1990; Perera-Castro et al. 2020). A significant point concerning temperature relationships is the broadly based curves described for most studies to date, with low but positive net assimilation over a wide range of temperatures, including 0 °C in some cases (Longton 1988).

A revision of optimum temperatures for photosynthesis in mosses around the world also revealed a general high optimum temperature with little evidence of latitudinal variation (Perera-Castro et al. 2022a). These results seem to differ from the measurements of relative growth rate when non-Antarctic mosses are grown under controlled conditions with an unnatural photoperiod of 12 h and no thermal oscillation between day and night. In such conditions an average optimum temperature for growth of 19 °C had been reported, with long-term temperatures of 30 °C being lethal for all studied mosses (Furness and Grime 1982a, b). However, when mosses are grown under more natural temperature regimes, with night temperatures lower than day temperatures, reported relative growth rates are significantly higher (Perera-Castro et al. 2022a). This suggests that the inhibition of night respiration by shortening night length or by exposure to low night temperatures is crucial for positive carbon balance of mosses. This is particularly relevant in Antarctica, where mosses currently experience temperatures higher than 15 °C for only 3% of their growing summer season (Perera-Castro et al. 2020). Therefore, the frequent characterisation of mosses as being inherently better adapted to cold conditions than angiosperms (Glime 2007) may be more related to an ability to inhibit respiration at low temperatures, rather than having lower optimum temperatures for photosynthesis. A question arises as to whether bryophytes present generally high Q_10_ values for respiration (change in rate of reaction per 10 °C change in temperature) or if Q_10_ could be acclimated to Antarctic environments, as has been shown in alpine vascular plants (Larigauderie and Körner 1995).

This means that a better understanding of how respiration responds to rising temperatures is required in order to model the effect of different climate change scenarios on the long-term net CO_2_ assimilation of Antarctic mosses and their survival. Light-saturated net CO_2_ assimilation of Antarctic cosmopolitan *Bryum argenteum* increases under a short, simulated heat wave (Gemal et al. 2022), although the long-term effect on carbon budget of an increase of respiration rates during warmer nights must also be considered.

### Microclimates

Antarctic mosses display characteristics that dramatically alter the Antarctic climate at micro scales to benefit their survival and productivity. In addition, the photosynthetic optimum temperatures closely resemble the microclimate conditions generated within the moss turfs (Longton 1988; Lewis Smith 1999; Perera-Castro et al. 2020; Randall 2022). This suggests that microclimate conditions within the moss turf provide a buffer from the extreme Antarctic climate, and as such, are extremely biologically and ecologically relevant (Melick and Seppelt 1997, Convey et al. 2014; King 2017; Robinson et al. 2018).

Temperature, light and water are all limiting factors at both high and low levels for Antarctic mosses (Adamson et al. 1988; Kappen et al. 1998; Robinson et al. 2000; Schlensog et al. 2004; Wasley et al. 2012; Robinson and Waterman 2014; Cruz de Carvalho et al. 2017; Perera-Castro et al. 2020, 2021). As such, periods when these factors are at intermediate levels are likely the times when Antarctic mosses experience the highest net photosynthesis and the least abiotic stress (Lewis Smith 1999; Perera-Castro et al. 2020, 2021). The freezing point of mosses has been shown to range between approximately −3 and −8 °C, differing between species or at different states of moss health (related to soluble carbohydrate content; Melick and Seppelt 1992). However, once frozen, they enter a state of physiological dormancy (Kappen and Schroeter 2002), such that they are typically unaffected by ambient temperature, light or water conditions. Outside of these dormancy periods when mosses are thawed (above their respective freezing points), photosynthesis can resume at canopy temperatures as low as 5 °C (Lewis Smith 1999; Perera-Castro et al. 2020) which can occur whilst air temperatures are as low as −2 °C (Randall 2022). This difference occurs through strategies that alter the microclimate in the moss canopy. Such strategies function as avoidance mechanisms of cold extremes and desiccation, and provide the moss with improved conditions for growth and productivity.

**Fig. 4:**
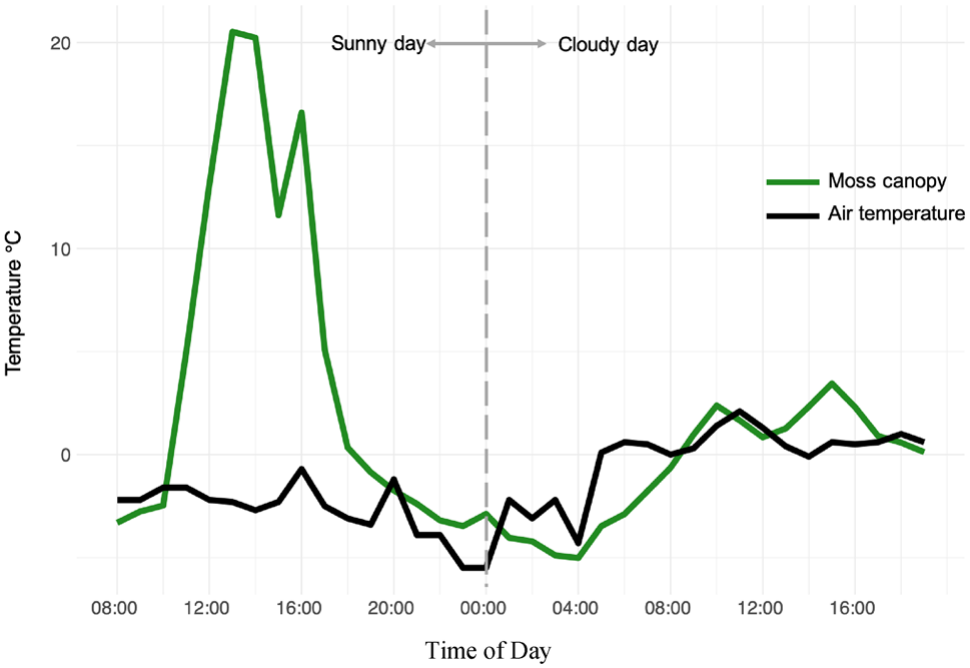
Diel hourly time series of Antarctic moss canopy temperature and weather station air temperature on a sunny day followed by a cloudy day. Moss canopy temperatures (*n* = 36) were measured using a thermocouple wire inserted 2 mm into the photosynthetic canopy of *Schistidium antarctici* near Casey Station in East Antarctica on two consecutive days, 7th–8th February 2022, with 3.5 h of darkness overnight separating the civil twilight of dusk and dawn. Air temperature observations were obtained for the same time period from the Casey Station automated weather station (AWS). All data were measured in Australian Eastern Daylight Time (AEDT, UTC+11) at Casey Station which is approximately 4 h ahead of solar time. Plotted data were adjusted for this time shift to align with solar time (UTC+7). Details of methodology for data collection are provided in Supplementary Information

**Fig. 5:**
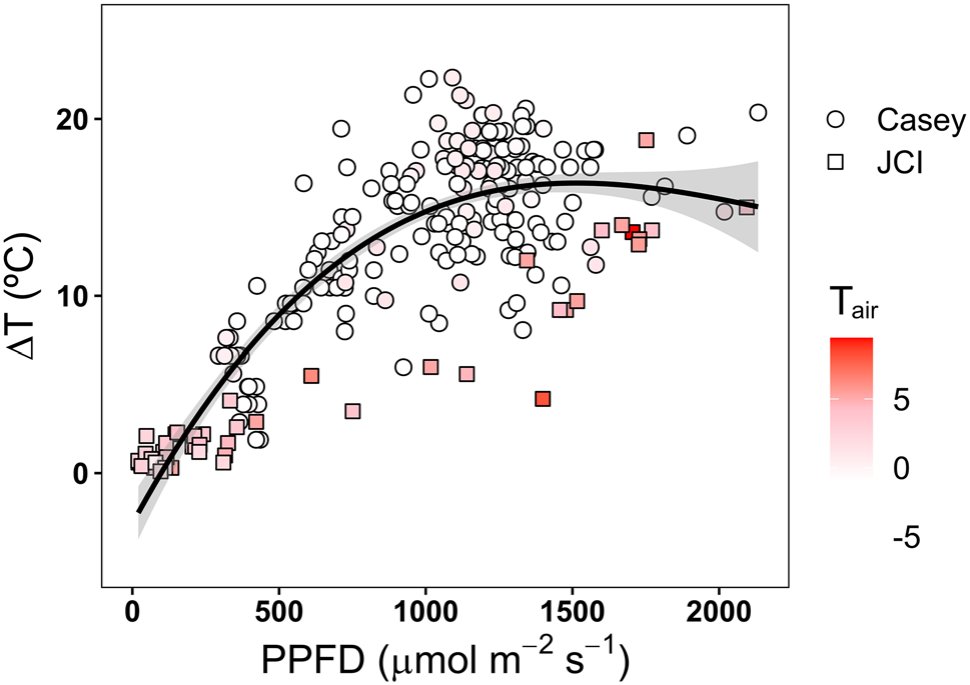
Difference between moss surface temperature and air temperature (ΔT) as a function of photosynthetic photon flux density (PPFD). Symbol shading denotes bins of air temperatures (< −5 °C, 0 °C, −5–0 °C, 0–5 °C and > 5 °C). The difference in temperature is lowest for the highest air temperatures. Data for Casey and JCI Station combined from Perera-Castro et al. (2020, 2021)

The development of favourable microclimates is achieved by individual and community level strategies aimed at maximising heat accumulation and minimising heat losses. At the individual gametophyte level, hydrated moss leaves are structured in such a way to increase the surface area available to absorb sunlight. On top of this, dark pigmentation of moss leaves reduces the albedo of the individual leaves and the moss canopy as a whole, therefore increasing the absorption of sunlight and subsequent heat gain (Malenovský et al. 2015). At the community level, densely packed gametophytes create a turf structure that reduces heat losses by slowing the turbulent transfer of air and heat in the air spaces immediately surrounding leaves in the moss canopy. This structure also reduces heat and water loss through evaporation. As a result, these strategies create a microclimate of warmer, humid air surrounding the moss leaves. Through these mechanisms, Antarctic mosses can achieve canopy temperatures of 20–30 °C in full sunlight when ambient air temperatures are close to 0 °C (Fig. 4 & 5; Longton and Holdgate 1967; Lewis Smith 1999; Perera-Castro et al. 2020; Gemal et al. 2022), especially on north and east facing topographic or micro-topographic aspects (Randall 2022).

**Fig. 6:**
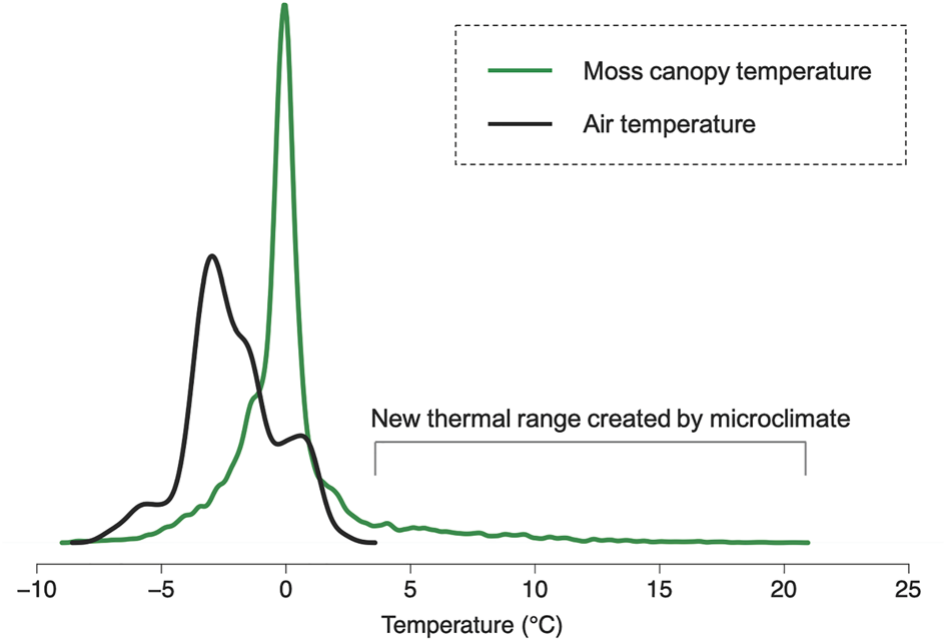
Density plot of Antarctic moss canopy temperatures and weather station air temperatures. Moss canopy temperatures (*n* = 13,856) were measured at 30 min intervals using thermocouple wires inserted 2 mm into the photosynthetic canopy of *Schistidium antarctici*, *Ceratodon purpureus* and *Bryum pseudotriquetrum* at *n* = 20 positions across moss beds in ASPA135 near Casey Station, East Antarctica, between 6th and 22nd February, 2022. Air temperature observations were obtained at 30 min intervals for the same time period from the Casey Station automated weather station (AWS). Details of methodology are provided in Supplementary Information

By modifying the microclimate within the moss canopy, mosses create a climate that differs from the broader Antarctic climate (Fig. 6; Longton and Holdgate 1967; Lewis Smith 1999). Over the summer growing season air temperatures display a relatively narrow range with temperatures typically at-or-below zero (Fig. 6). However, temperatures measured in the moss canopy show considerably higher maximum temperatures, up to 20 °C (Fig. 6), closely resembling optimum photosynthetic temperatures (Perera-Castro et al. 2020). Importantly, these warm microclimate conditions constitute new thermal climate conditions outside of the conditions provided by the broader climate (Fig. 6), extending the thermal range of the environment and providing opportunities for optimum photosynthesis that otherwise would not occur (Pannewitz et al. 2005; Gemal et al. 2022; Randall 2022). However, these maximum temperatures only occur for short windows of time when the mosses are in direct sunlight (Longton 1974; Pannewitz et al. 2003), whereas most of the time photosynthesis is likely to be greatly depressed by cold temperatures (Kappen and Schroeter 2002; Pannewitz et al. 2003; Perera-Castro et al. 2020; Gemal et al. 2022).

Warming to these temperatures is primarily driven by direct insolation (Longton and Holdgate 1967; Longton 1974; Kappen et al. 1998; Perera-Castro et al. 2020; Baker et al. 2021; Gemal et al. 2022; Randall 2022). Therefore, the ability for mosses to accumulate heat is heavily impacted by cloud and/or snow cover, which can inhibit mosses from warming above air temperatures (Fig. 4; Longton and Holdgate 1967), and limit opportunities for photosynthesis. Antarctica experiences some of the steepest shifts in seasonal solar angles and daylight hours worldwide (Pannewitz et al. 2005; Convey et al. 2014; Gemal et al. 2022). Solar elevation (zenith) angles are much lower to the horizon compared to lower latitudes, reducing the quantity of light that reaches the ground where Antarctic mosses grow. However, freeze–thaw cycles cause moss turfs to develop lumpy (West Antarctica) or ridged (East Antarctica) micro-topography (Fig. 1; Melick and Seppelt 1992; Lewis Smith 1999; Lovelock and Robinson 2002; Porada et al. 2016; Park et al. 2018) that can position mosses perpendicular to incoming solar radiation, maximising the light that they receive (Randall 2022). Consequently, Antarctic mosses can receive exceptionally high light quantities, often exceeding 1000 µmol m^−2^ s^−1^ and sometimes reaching 2000 µmol m^−2^ s^−1^ (Pannewitz et al. 2003; Convey et al. 2014; Perera-Castro et al. 2020; Gemal et al. 2022; Randall 2022). It is during these higher intensities of solar insolation that mosses reach their warmest canopy temperatures (Fig. 5).

Whilst warmer temperatures facilitate higher photosynthetic rates, the thermal regime in the moss canopy is strongly governed by water content (Lewis Smith 1999). The interactive effect of temperature and water content can have different outcomes for mosses connected to summer melt streams and those relying solely on melt after snowfall. In areas where moss receive a reliable flow of water, warming (and cooling) of the moss canopy is strongly inhibited by the buffering effect of water (Lewis Smith 1999; Pannewitz et al. 2003, 2005; Block et al. 2009; Perera-Castro et al. 2022b). Under these conditions, moss canopy temperatures are typically either in equilibrium with water temperatures or closely linked to water temperatures (Pannewitz et al. 2003), depending on the level of saturation. For these mosses, the risk of desiccation is minimal. However, photosynthetic rates are likely to be depressed by the cooling effect of the water (Pannewitz et al. 2003), or possibly through oversaturation (Perera-Castro et al. 2020).

Conversely, in areas where mosses are distributed away from a continuous water source, they typically rely on melt from overlying snow after a snowfall event. The transmission of light through the overlying snow can reach the underlying moss and warm the moss canopy, depending on the snow depth. Warmth from the moss canopy can then melt the overlying snow from underneath, providing liquid water (Melick and Seppelt 1992). Once mosses are hydrated, their leaves are oriented to maximise light absorption, further aiding in the warming and melting of the snow. Remarkably, whilst the snow is being melted from underneath, the remaining snow cover can provide an “icehouse” effect (Schroeter et al. 2021), allowing light to enter whilst providing protection from dry ambient air, wind and desiccation (Körner 2003). There is also evidence that suggests that warmer temperatures may facilitate the upward wicking of water from the ground as a source of hydration for mosses (Noakes and Longton 1988). The ability to melt snow from beneath or wick water from the ground are especially important for mosses not connected to melt streams, ponds or lakes, as they may be the dominant mechanisms for obtaining liquid water over the growing season. However, just as warm temperatures can help to provide liquid water, warm temperatures are also associated with increased rates of water loss through evaporation. For mosses exposed to free air and not connected to continuous melt water, warmer canopy temperatures represent greater risks of desiccation and reduced photosynthetic capacity (Kappen et al. 1998; Raggio et al. 2016; Randall 2022) and a greater need for photoprotection.

### Protection from excess sunlight

Field studies show photosynthesis is well protected from excess solar radiation, partly through increases in protective pigments (Searles et al. 2001; Newsham and Robinson 2009). Protective carotenoids, including xanthophylls and β-carotene, commonly increase in response to high photosynthetically active radiation (Esteban et al. 2015), and in Antarctic plants exposed to elevated UV-B radiation (UV-BR, 280–315 nm) (Newsham 2003; Newsham et al. 2002; Robinson et al. 2005; Ruhland and Day 2001).

Whilst light is necessary for photosynthesis to proceed, both visible and UV-BR wavelengths can damage the photosynthetic apparatus, either directly or through the production of reactive oxygen species (ROS; Takahashi and Badger 2011; Badmus et al. 2022). Other stressors, such as cold and drought, exacerbate damage by slowing the enzymatic reactions of carbon fixation and protective processes such as photorespiration, thus reducing electron transport (García-Plazaola et al. 2012; Takahashi and Murata 2008). Destructive ROS can form in photosystem II (PSII), if absorbed light energy is not quickly passed into electron transport (Takahashi and Badger 2011). These ROS potentially oxidise components of the photosynthetic apparatus, such as chlorophyll (Takahashi and Badger 2011). Carotenoids, including β-carotene and xanthophyll pigments can mitigate such damage through ROS scavenging (Havaux et al. 2007), and additionally the latter pigments play an important role in dissipating excess light energy as heat in a process called non-photochemical quenching (NPQ).

Within the xanthophyll cycle (VAZ), violaxanthin (V) is enzymatically converted to zeaxanthin (Z) via antheraxanthin (A) (Demmig-Adams et al. 1990, 2012, 2020; Nichol et al. 2012). In high light Z can form within 5–15 min, but will only dissipate light as heat when high light creates a pH gradient (∆pH) across the thylakoid membrane. This system is rapidly responsive to fluctuating light levels; as light levels ease, ∆pH relaxes and photosynthetic efficiency is rapidly restored, even whilst Z is still present (Niyogi et al. 2005; Demmig-Adams et al. 2012; Gerotto et al. 2012). Conversion of Z back to V is a slower process, occurring overnight in darkness in most ecosystems (Demmig-Adams et al. 2012). Mosses possess two Light-Harvesting Complex superfamily proteins which function in NPQ; retaining the Light-Harvesting Complex Stress-Related (LHCSR) proteins found in algae and the Photosystem II Subunit S (PSBS) common in vascular plants (Pinnola 2019; Pedraza-González et al 2023).

The VAZ pool size, as well as Z concentrations increase either in high light or with other environmental stressors that exacerbate light damage to PSII (Takahashi and Murata 2008). Certain stressors, such as cold and drought, can result in sustained Z accumulation. Overwintering conifers have sustained Z concentrations, not reversed in overnight darkness but by warming; and desiccation tolerant cryptogams (like Antarctic lichens and mosses) appear to accumulate Z during desiccation (Fernández-Marín et al. 2010, 2011; Verhoeven 2014, 2013). Since mosses such as *C. purpureus* lose cellular water as they freeze, desiccation and freezing stress may be indistinguishable (Verhoeven 2014; Lenné et al. 2010). Reversible photoinhibition occurs in Antarctic bryophytes during freeze–thaw cycles and in high light at both low and high temperatures (Lovelock et al. 1995a, 1995b; Kappen et al. 1989; Adamson et al. 1988).

Antarctic mosses acclimate their pigment concentrations to seasonal changes and between sites presumably in response to microclimate variation (see above) (Schroeter et al. 2012; Snell et al. 2007; Lovelock and Robinson 2002; Robinson et al. 2005; García-Plazaola et al. 2022). When mosses are covered by ice and snow during the long winter, their pigments adjust to cope with the shaded environment by reducing photosynthetic rates and carotenoid concentrations whilst increasing chlorophyll levels (Post 1990; Post and Vesk 1992; Robinson et al. 2005). However, in the austral summer, mosses lose their protective cover and are exposed to high light stress, compounded by cold temperatures and desiccation events. At this point, the percentage of VAZ sustained as Z is usually high in Antarctic mosses (Lovelock et al. 1995b; Lovelock and Robinson 2002; Martínez-Abaigar and Núñez-Olivera 2022; García-Plazaola et al. 2022), similar to that of sun plants (Demmig-Adams and Adams III 1992; Lovelock and Robinson 2002; García-Plazaola et al. 2022). For instance, when *S. antarctici* samples were moved from the field into the laboratory, 50% of the VAZ pool remained as A+Z after 24 h in low light (Lovelock et al. 1995a). Therefore, the pigment concentrations of Antarctic mosses are highly agile to changing environmental conditions, enabling them to acclimate and thrive in extreme conditions.

**Fig. 7:**
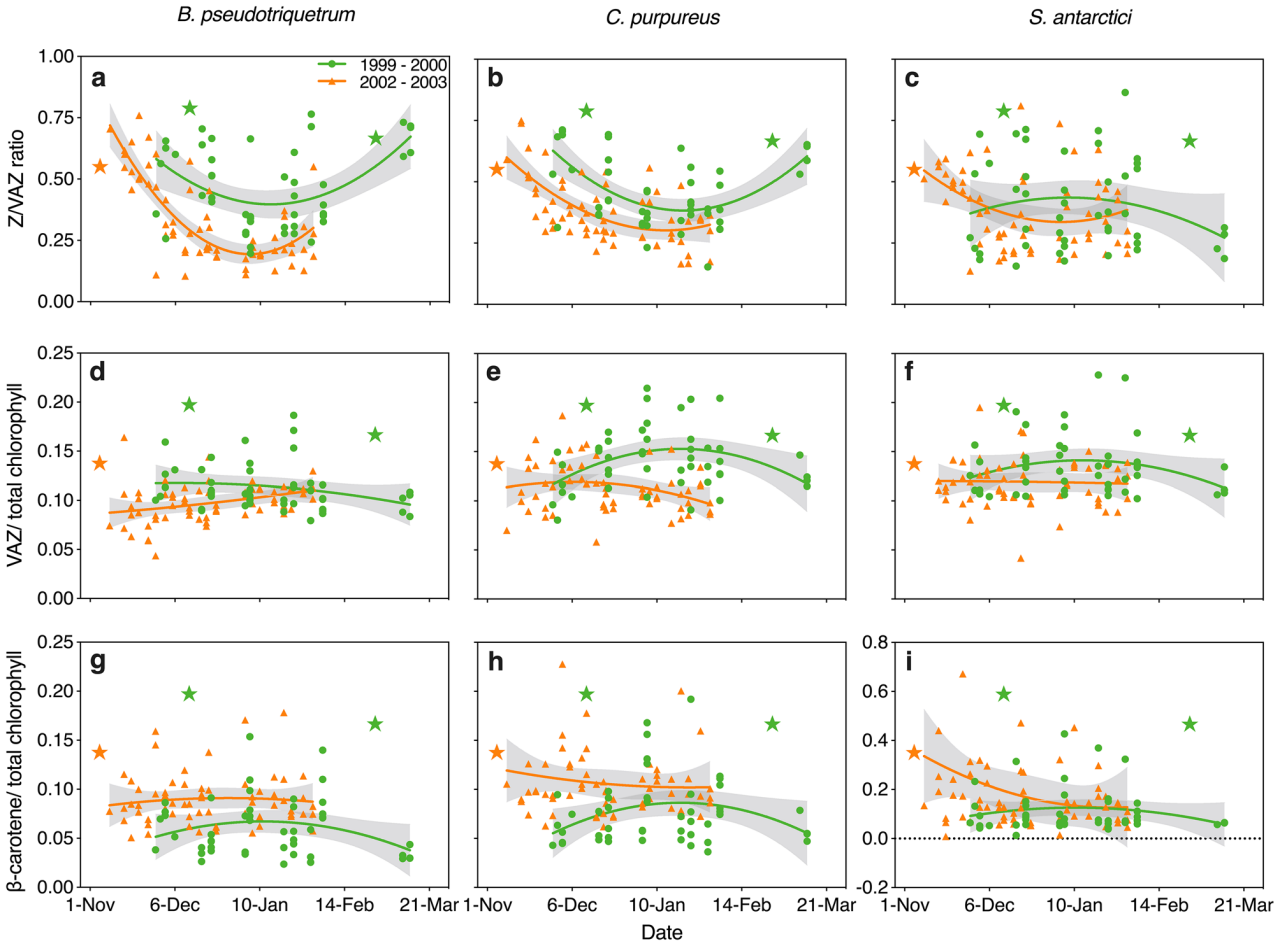
Changes in photoprotective carotenoids in Antarctic mosses over two contrasting summer seasons (1999–2000 and 2002–2003) in the Windmill Islands Antarctica. Variation in the proportion of xanthophyll cycle present as **a﻿–c** zeaxanthin (Z/VAZ,), **d–f** VAZ/total chlorophyll and **g–i** β-carotene/total chlorophyll ratio in three moss species *Bryum pseudotriquetrum*, *Ceratodon purpureus* and *Schistidium antarctici* (N.B. carotenoid/chlorophyll ratios are expressed as mmols.mol^−1^)*.* Second order polynomial regressions are displayed as solid lines and their 95% CI as shade whenever significant regressions were found. The 1999–2000 season (*n* = 47 for each species) exhibited strong ozone depletion and relatively low ‘summer melt’ (see Fig. 2). The growing season started on 11th December and ended on 27th February, with temperatures exceeding 0 °C for 41 days. Over the season, ozone layer thickness oscillated about a mean of 313 Dobson Units (DU). A minimum ozone layer thickness of 187 DU occurred on 5th October with a maximum thickness of 427 DU on 17th October. Conversely, the 2002–2003 summer growing season (*n* = 64 for each species) had atypical ozone depletion with anomalous and widespread melt. The summer season began on 4th November and ended on 13^th^ March, with temperature exceeding 0 °C for 68 days. The seasonal mean ozone column depth above Casey was 339 DU. The minimum and maximum ozone depth for the season were 260 DU and 440 DU on 30^th^ January and 20^th^ February, respectively. The star symbols indicate the start and end of the melting period in the growing season, except for the 2002–2003 season where the endpoint is unmarked due to limited sampling time. Further details of climate and sample collection are provided in Dunn and Robinson (2006) and Turnbull et al. (2009). For extraction methodology see Supplementary Information

Changes in these protective pigments within three moss species at Casey Station were determined over two contrasting summer growing seasons (Fig. 7; Dunn and Robinson 2006; Turnbull et al. 2009). Here we focus on the xanthophyll cycle pigments because of their role in NPQ, and β-carotene which is the precursor of the xanthophyll pigments and can act as an effective antioxidant to directly neutralise reactive free radicals. The 1999–2000 summer season was characterised by high ozone depletion, high UV-BR and relatively low summer melt with temperatures exceeding 0 °C on just 41 days between November and March (Fig. 2c). In 2002–2003 anomalous ozone depletion (Varotsos 2002) was accompanied by 68 days above 0 °C and widespread melt (Fig. 2c).

**Fig. 8:**
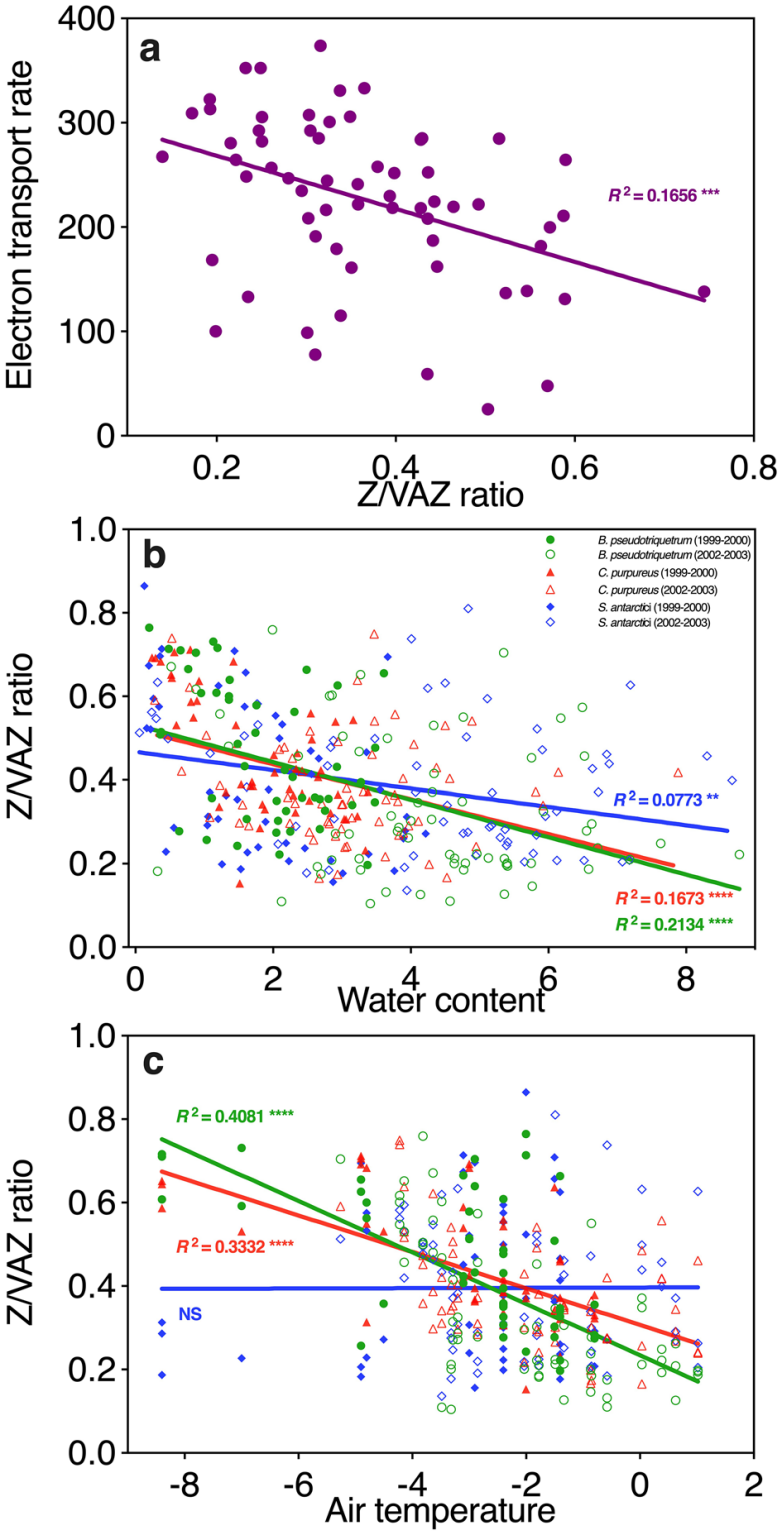
Relationship between photoprotective xanthophyll pigments (Z/VAZ) in Antarctic mosses relative to photosynthetic electron transport rate measured by chlorophyll fluorescence (*n* = 64) (**a**), moss water content (gH_2_O gdw^−1^) (**b)**, and air temperature (°C) (**c)**. Associations for three moss species, *B. pseudotriquetrum* (*n* = 111, green)*, C. purpureus* (*n* = 110, red) and *S. antarctici* (*n* = 108, blue) **b–c** measured across two seasons are shown, however ETR data in **a** were only collected in 2002–2003 and all three species showed a similar response

Zeaxanthin was present throughout the season in all species suggesting sustained Z accumulation (Demmig-Adams et al. 2012, 2020; García-Plazaola et al. 2012; Verhoeven 2014). Retention of Z is advantageous in a predictably cold climate since NPQ can then be activated rapidly (Demmig-Adams et al. 2012, 2020). This is important since even in mid-summer temperatures can still plunge below zero overnight (Fig. 2a). Sustained higher overall levels of Z/VAZ were apparent in the more stressful environmental conditions in 1999–2000 (colder, drier and more UVR) than the more benign growth conditions in 2002–2003. High rates of *Z* conversion were shown to correlate with reduced electron transport (ETR) in 2002–2003 (*R*^2^ = 0.1656, *p* < 0.001 Fig. 8a).

The seasonal Z/VAZ (Fig. 7) suggests that both *B. pseudotriquetrum* and *C. purpureus* have a high capacity to quench excess light throughout the summer (Fig. 7a, b), with similar trends in both seasons. The proportion of Z/VAZ was highest (> 60%) early in both seasons, but also late in the season in 1999–2000. In mid-summer when daylength approaches 24 h and conditions are relatively warm and wet, the Z/VAZ declined to more moderate levels (about 40% in 1999–2000). The more favourable conditions in 2002–2003 resulted in greater reconversion of zeaxanthin, from 70 to 14% of the pool in *B. pseudotriquetrum* and from 59 to 24% in *C. purpureus* (Fig. 7). In contrast, the endemic moss species, *S. antarctici* showed a lot of mid-season variability, with sustained high levels early in the season and consistently low levels at the end. This may reflect the differences in distribution and preferred microhabitat between these three species with *B. pseudotriquetrum* and *C. purpureus* occupying more extreme microsites than *S. antarctici* (Robinson et al. 2018; Wasley et al. 2006).

The VAZ/chlorophyll ratio did not show strong seasonal trends, with variability probably reflecting differences between microsites. The only seasonal trend that was significant was an increase during mid-summer in *C. purpureus* in the more stressful 1999–2000 season (*P* < 0.05). Similarly, the β-carotene/chlorophyll showed no seasonal trend in *B. pseudotriquetrum* or *C. purpureus* but was much more variable in *S. antarctici* (Fig. 7). There appears to be more β-carotene/chlorophyll in the latter species, than in the cosmopolitan species and levels were highest early in the season and least at the end of the season.

The environmental drivers for these seasonal changes in pigments were also investigated. Where similar data collection allowed (namely air temperature and water content), data were combined for both seasons. We selectively report the robust, consistent trends across seasons, acknowledging that the R^2^ are low. (Fig. 8; Supplementary Fig. S1). Correlations for UV index are also shown in Supplementary Fig. S2, but these were weaker than water content and air temperature and common to only one species. The proportion of the xanthophylls in the protective form (Z/VAZ) decreased as moss became wetter in all three species (Fig. 8b), consistent with dry moss needing most photoprotection, and also greater enzymatic activity under wetter conditions. In *S. antarctici* there was also a negative association between VAZ/chlorophyll and water content (*R*^2^ = 0.0486, *P* < 0.05, Supplementary Fig. S1). Contrastingly, there was a positive association between β-carotene/chlorophyll and water content in both *B. pseudotriquetrum* and *C. purpureus* (*R*^2^ = 0.1271, *P* < 0.001 for *B. pseudotriquetrum*, *R*^2^ = 0.0697, *P* < 0.01 for *C. purpureus*, Supplementary Fig. S1).

The proportion of the xanthophylls in the protective form (Z/VAZ) decreased under warmer conditions in *B. pseudotriquetrum* and *C. purpureus* (*R*^2^ = 0.4081, *P* < 0.0001 for *B. pseudotriquetrum*, *R*^2^ = 0.3332, *P* < 0.0001 for *C. purpureus*), but not *S. antarctici* (Fig. 8c)*.* β-carotene/chlorophyll was positively associated with air temperature for *B. pseudotriquetrum* (*R*^2^ = 0.0771, *P* < 0.01), but not for the other two species (Supplementary Fig. S1)*.*

Often air temperature and water parameters change together, e.g. freezing reduces moss water content, but moss can also be dry when it is relatively warm. Taken together these data seem to indicate that water content is the stronger driver. These relationships likely explain the trends shown in Fig. 7, with drier moss (early and late in the season), having to dissipate more excess light than the wetter, warmer moss in mid-summer and thus needing more zeaxanthin (and in the case of *S. antarctici* also more total xanthophyll cycle pigments relative to chlorophyll). This is also consistent with the data for all species showing that Z/VAZ was lowest when electron transport rates were highest (Fig. 8a; 2002–2003 season only).

The endemic species, *S. antarctici,* differed from the other two species by showing low values of Z/VAZ at the end of the season and no response to air temperature. This may be explained by it occupying the lowest sites and possibly maintaining hydrated status for more of the season. *Schistidium antarctici’s* response to air temperatures might also be explained by a buffering effect of wet moss, since the moss canopy temperature range experienced may be less extreme for this species. Further studies of pigment responses across microclimates are needed to resolve this.

### Ultraviolet radiation and the Antarctic ozone hole

In mid-summer high levels of photosynthetically active radiation, naturally brings higher levels of UV radiation. However, reduction of stratospheric ozone directly above Antarctica presents another challenge for mosses living in the unique Antarctic climate. The layer of ozone molecules in the stratosphere is one of the Earth’s defences against harmful solar radiation, particularly the shorter wavelengths such as UV-B (280–315 nm) and UV-C (100–280 nm) light. Since the 1970s, catalytic breakdown of ozone molecules has occurred during the austral spring (September to November) due to human-made chlorine-based aerosols reaching the stratosphere (Bernhard et al. 2023). This has depleted the ozone layer and reduced its effectiveness as a UV filter, subjecting Antarctic mosses to elevated and harmful doses of UV-B radiation; levels of which can impair vital cellular contents and processes, such as DNA, chlorophyll and photosynthesis (Rozema et al. 2005; Seppelt et al. 2011; Newsham and Robinson 2009). Considering their lack of structural defences, what mechanisms do Antarctic mosses employ to survive under elevated UV-B radiation?

Mosses are generally well protected from UV-A and UV-B radiation by their production and storage of specialised compounds called UV-absorbing or -screening compounds (Newsham and Robinson 2009; Robinson and Waterman 2014), and by activation of antioxidative enzymes and DNA repair processes (Martínez-Abaigar and Núñez-Olivera 2022; Wang et al. 2021). It is well documented that several Antarctic species utilise UV-absorbing compounds as a direct protection mechanism to absorb harmful UV wavelengths and transmit useful visible light for photosynthesis to the chloroplasts (Newsham and Robinson 2009; Davies et al. 2020; Waterman et al. 2018, 2017; Dunn and Robinson 2006; Newsham 2003; Newsham et al. 2002; Clarke and Robinson 2008). UV-absorbing compounds appear to protect Windmill Islands species from the DNA damage expected under elevated UV-B radiation, especially when such plants are desiccated (Clarke and Robinson 2008; Turnbull et al. 2009).

Derivatives of these screening compounds in Antarctic bryophytes are mainly phenolic or flavonoid based (Waterman et al. 2017; Ryan et al. 2009; Snell et al. 2009; Newsham 2003; Webby et al. 1996; Markham and Given 1988; Davies et al. 2020), and several have antioxidant properties with the capacity to also mop up ROS within the cell (Martínez-Abaigar and Núñez-Olivera 2022; Robinson and Waterman 2014); indirectly preventing further damage. Other antioxidative mechanisms, such as activation of antioxidative enzymes, can also occur within Antarctic mosses, e.g. *Pohlia nutans* and *Sanionia uncinata*, to help quench ROS formed under stressful conditions like excess light and desiccation (Martínez-Abaigar and Núñez-Olivera 2022; Pizarro et al. 2019; Li et al. 2019). Some moss species, including *C. purpureus*, also place effective UV-absorbing compounds in their cell walls, providing a better defence strategy for these single celled organisms than when placed in the vacuole (Clarke and Robinson 2008; Waterman et al. 2017). Antarctic bryophyte species can exhibit reddish pigmentation due to the accumulation of UV-absorbing compounds (Fig. 3c; Newsham 2010; Snell et al. 2009; Waterman et al. 2018). There is evidence that sunscreens like flavonoids may accumulate in mosses under desiccating, nutrient deprivation and extreme temperature conditions in combination with UV radiation (see review Martínez-Abaigar and Núñez-Olivera 2022; Davies et al. 2020), and that they can be used as indicators of moss health (Waterman et al. 2018; Malenovský et al. 2017) and as climate proxies in Antarctica (Markham et al. 1990; Ryan et al. 2009).

### Fertiliser from ancient penguin colonies

Antarctic soils are often poorly developed and relatively nutrient poor, with nutrients mostly provided from seabirds and mammals (Erskine et al. 1998) meaning that vegetation is often located adjacent to nesting sites (Bokhorst et al. 2019). In extant penguin colonies nutrient loads are too high for most plants and these sites are also subject to trampling (Cannone et al. 2022). Ancient penguin colonies, however, provide a rich source of weathered guano and the Casey region’s rich moss beds are found in areas where penguins nested 3000–8000 years ago (Fig. 3a). With glacial retreat since the last ice age, isostatic uplift has lifted the land up and the penguins have moved to sites offshore on nearby islands. The abandoned Adélie penguin colonies are marked by carefully graded rocks of nest pebbles, and guano that provides nutrients for the moss beds (Fig. 3b). Radiocarbon dates on penguin bone and eggshell confirm the age of this freeze-dried fertiliser (Goodwin 1993; Emslie and Woehler 2005), and stable isotopes of nitrogen can be used to show that present day mosses derive nutrients from this guano supplemented by smaller amounts of airborne ammonium from current penguin colonies (Wasley et al. 2012).

### The future of Antarctic moss beds

Climate change is happening in Antarctica, driven by both increasing greenhouse gases and ozone depletion (Robinson and Erickson 2015; Chown et al. 2022; WMO 2018, 2022; Ranasinghe et al. 2021; Fox-Kemper et al. 2021; Constable et al. 2022). The peninsula and western Antarctica have experienced rapid warming including reductions in ice cover which opens up new land for colonisation (Lee et al. 2017; Cannone et al. 2022; Colesie et al. 2023). The eastern side of the continent has remained cooler, in part due to ozone depletion (Robinson and Erickson 2015; WMO 2018) but future warming of continental Antarctica is predicted (Chown et al. 2022; Ranasinghe et al. 2021; Constable et al. 2022). This will open up new areas for moss colonisation but as discussed in Lee et al. (2022b), it is still not clear if this will be beneficial to all species. A lot will depend on whether the new ice-free areas maintain a good supply of water and if nutrients are present. Even if conditions are favourable, propagules have to disperse into new areas and establishment can be threatened by disturbance (Lee et al. 2022b; Cannone et al. 2022).

Climate change has resulted in net snow accumulation over much of Antarctica and increasing air temperatures in maritime Antarctica (Gutiérrez et al. 2021; Ranasinghe et al. 2021; Constable et al. 2022). Increased wind speeds have occurred over the southern ocean especially in the austral summer (Chown et al. 2022; WMO 2018). Ozone depletion has increased the levels of UV-B radiation incident over Antarctica, with the early summer UV index recently shown to exceed those measured at mid latitudes (Bernhard et al. 2023). In recent decades increased greenhouse gas concentrations and ozone depletion have resulted in a poleward shift in the westerly jet stream associated with an increasingly positive phase of the Southern Annular Mode (SAM) climate pattern (Abram et al. 2014; Robinson and Erickson 2015; WMO 2018, 2022; Chown et al. 2022). Wind affects water supply in many ways from blowing snow, to evaporating water once it is melted.

Extreme climatic events are also becoming more common in Antarctica. In 2019/20, anomalous high temperatures were recorded across Antarctica throughout the summer, bringing new maximum temperature records (18.3 °C, Robinson et al. 2020; Wille et al. 2019). In autumn 2022, an extensive heatwave across Antarctica brought unseasonably warm temperatures for autumn and additional precipitation (Barnes et al. 2023).

**Fig. 9:**
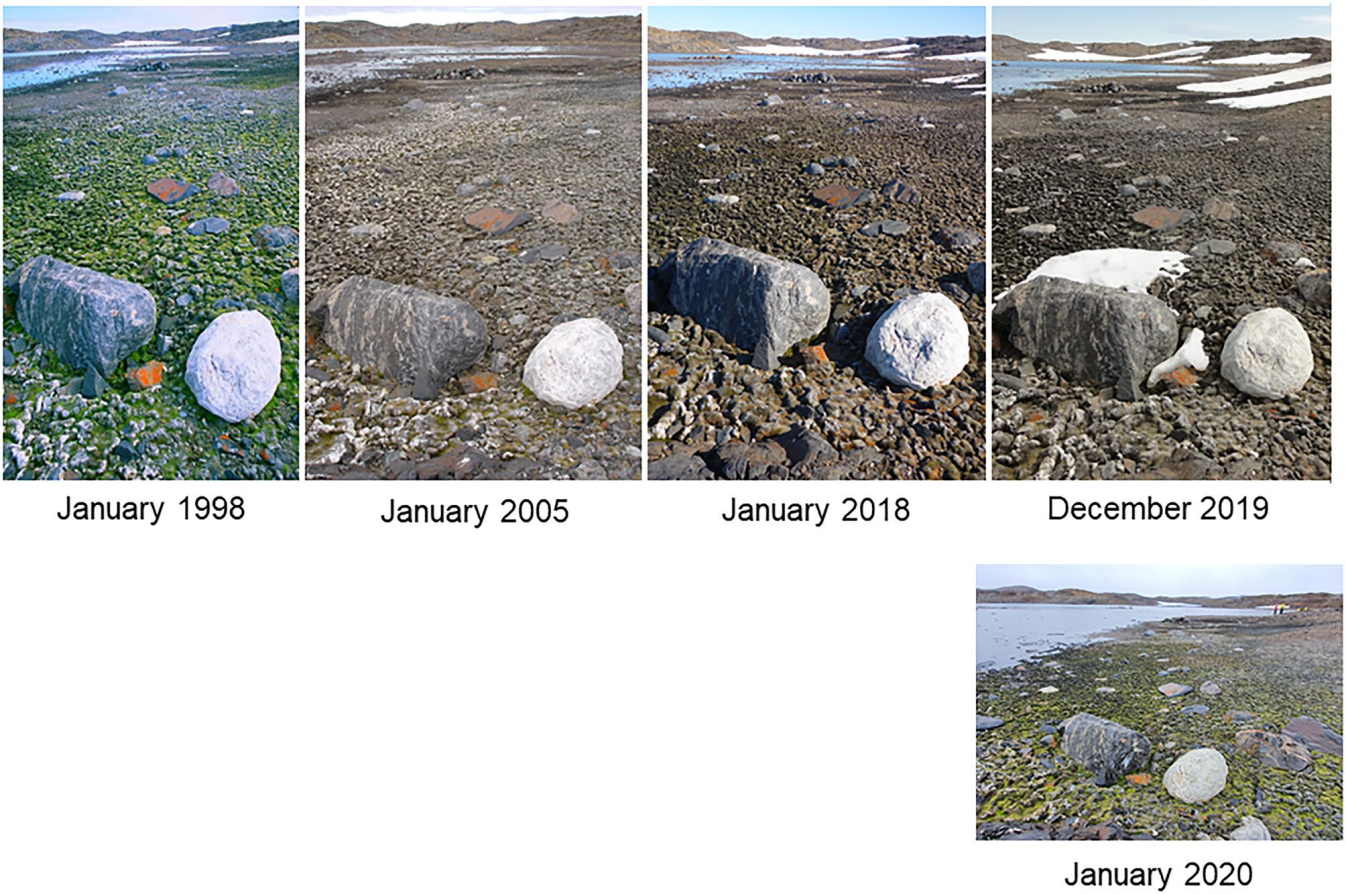
Decline in moss health from healthy (1998) to moribund (2005, 2018 and Dec. 2019) at Mossell Lake, Vestfold Hills, Antarctica presumably because of reduced water supply to a former extensive moss bed surrounding a lake, which was previously filled by glacial melt. Bottom panel (Jan. 2020) shows some repair occurred after flooding in the 2020 summer heatwave. (Images John French, Marcus Salton, Dana Bergstrom; modified with permission from Bergstrom et al. 2021)

Ozone depletion and global heating have both contributed to a drying trend in the Windmill Islands over the last half century (Robinson et al. 2018). Observations from another site thousands of kilometres to the West, Mossell Lake in the Vestfold Hills (Fig. 9), suggest long-term drying of large moss beds may be more widespread. The future trajectory of these Antarctic moss beds is obviously linked to how future climate changes and especially how this affects the water balance in East Antarctica (Guglielmin et al. 2014; Robinson et al. 2018; Bergstrom et al. 2021).

Whilst water availability is probably the dominant driver of terrestrial biodiversity patterns in Antarctica (Convey et al. 2014) it is relatively poorly resolved in future models for ice-free areas of Antarctica as well as for the Arctic (Constable et al. 2022). A key area for research into the future of Antarctic terrestrial ecosystems is what happens to water availability as ice-free areas expand (Lee et al. 2017, 2022b; Guglielmin et al. 2014; Cannone et al. 2016, 2022; Loisel et al. 2017; Torres-Mellado et al. 2011; Favero-Longo et al. 2012; Yu et al. 2016; Colesie et al. 2023).

Currently the major source of water is snow and ice banks which supply melt water over summer. Vegetation and invertebrate communities are often tied to these seasonal melt water sources. If there is more precipitation and it gets wetter we would expect increased growth and associated greening, as has been observed in maritime Antarctica (Amesbury et al. 2017; Royles et al. 2013; Cannone et al. 2022; Colesie et al. 2023). In East Antarctica where snowbanks retreat with warming, existing communities will have to shift to keep up with retreat if precipitation does not increase. Early onset of spring snow melt, with higher peak flows at the expense of summer flows, is a threat as identified in other snow dominated regions globally (IPCC 2021). In the McMurdo Dry Valleys intense glacial melt in the ‘2002 flood year’ produced a step-change in water availability which triggered distinct species-specific changes in cyanobacterial and invertebrate communities in the following years (Gooseff et al. 2017). Greening of a large, previously moribund moss bed in the Vestfold Hills, East Antarctica (Fig. 9) occurred during flooding precipitated by the 2020 heatwave (Bergstrom et al. 2021). In the Windmill Islands, large sections of moss have been dislodged from our long-term monitoring sites, presumably by similar flood events (Fig. 1n). This type of disturbance can enable movement of moss to new locations (Skotnicki et al. 1999) but can also result in loss of vegetation cover if moss is deposited in a new unfavourable location.

In this already extremely water-limited habitat, environmental factors that influence the supply of water will have a profound effect on moss distribution, growth and survival. Although we recognise that the future of Antarctic vegetation will depend on the availability of water this is probably the most uncertain factor in climate modelling. This is due to uncertainties in predictions of inputs (precipitation as snow and rain), poor understanding of local snow accumulation and its melt characteristics as well as how much water is retained in the ecosystem. Better models of precipitation and finer scale modelling of the microclimate and hydrology will aid our understanding of the role that water will play in the future. In addition to changes in direct inputs such as precipitation, temperature and radiation patterns both influence snow melt. Increased temperature, especially driven by strong radiation (Fig. 5), results in increased snow melt which will be positive (Fig. 9), providing it does not result in destructive flooding or drain too quickly (either through thawing permafrost; Guglielmin et al. 2014, or from the catchment). Increasing temperature and wind speeds also increase evaporation of water from moss beds resulting in less water for photosynthesis and growth. Increased evaporation can indirectly affect cloud cover (Mendoza et al. 2021), which would reduce the timing and quantity of radiation available to provide for photosynthesis, canopy warmth and water supply.

Ozone depletion reaches its maximum in spring (October) when the solar angle and thus radiation levels are low. In spring Antarctic mosses will also be protected by snow cover. However, in the past few years ozone depletion has extended into early summer and this has resulted in Antarctic measurements of quite extreme summer UV radiation (Bernhard et al. 2023). This is a worrying development because the timing coincides with the emergence of moss from under snow cover. It is ironic that whilst the Montreal Protocol (and its amendments) have been very successful and the ozone layer over Antarctica is starting to recover we are faced with a situation where Antarctica’s plants and animals may currently be exposed to some of the highest UV-B levels since ozone depletion was first observed in the 1970s. As noted above, generally Antarctic mosses seem well protected from UV-B radiation, but it does contribute to the combined environmental stress, and results in slightly reduced carbon gain (Newsham and Robinson 2009). The cumulative UV dose experienced by Antarctic plants will likely increase in future, if global heating and increased extreme heat events lead to early snow melt.

Physiological evidence shows Antarctic plant’s low stature and microclimate allows them to maintain surface temperatures well above air temperature (> 10 °C above ambient) and consequently they have metabolic optima similar to temperate plants (> 20 °C, Perera-Castro et al. 2020). This is an excellent adaptation to the current climate but we do not know how well moss will respond to the much higher temperatures that occur in heatwaves such as those experienced in the 2019–2020 summer (Robinson et al. 2020; González-Herrero et al. 2022). Will temperatures exceed limits and cause detrimental damage, or will mosses be able to avoid or cope with additional stress by using their suite of photoprotective compounds? A better understanding of the response of both photosynthesis and respiration to warmer temperatures is needed to model future growth rates of Antarctic mosses.

Some native organisms will be winners and some losers as competitive processes alter in this rapidly changing environment (Lee et al. 2022a, b). For example, in the Windmill Islands native, cosmopolitan moss species are shifting into areas previously dominated by endemic moss species (Robinson et al. 2018) and some species show enhanced ability to colonise newly disturbed areas around stations (e.g. *B. pseudotriquetrum* at Casey Station; Robinson *pers comm*). On the peninsula mosses and lichens are also vulnerable to expansion of native angiosperms, increased animal disturbance and increased risk of invasive species (Chown et al. 2022; Cannone et al. 2022; Bokhorst et al. 2019; Colesie et al. 2023).

Antarctic mosses exhibit a range of physiological strategies which have enabled them to maintain habitation in Antarctica despite its harsh conditions. Climate change is changing this environment and some of these adaptations may not be as favourable in future. Understanding the role of microclimates in ameliorating harsh conditions, the future characteristics of ice-free areas (Lee et al. 2022b) and the extent to which moss species can adapt is vital to accurately predict their future.

### Supplementary Information

Below is the link to the electronic supplementary material.

Supplementary file1 (DOCX 334 KB)

## Data Availability

Data sets are available via the Australian Antarctic Data Centre https://data.aad.gov.au/AAS_4046_TempOptima_Frontiers_Perera-Castro;ASAC_1313_Moss_Field_Measurements
