# Supplementary material for: Basking in the sun: how mosses photosynthesise and survive in Antarctica
Source: Photosynth Res. 2023 Jul 29;158(2):151–69. doi: 10.1007/s11120-023-01040-y (PMC10684656; doi:10.1007/s11120-023-01040-y)
Supplement: Supplementary file 1 — Supplementary file1 (DOCX 334 KB) [file 11120_2023_1040_MOESM1_ESM.docx]

Supplementary Information

Basking in the sun: how mosses photosynthesise and survive in Antarctica

Hao Yin^1,2^, Alicia V. Perera-Castro^3^, Krystal L. Randall^1,2^, Johanna D. Turnbull^1,2^, Melinda J. Waterman^1,2^, Jodie Dunn^1,2^ and Sharon A. Robinson^1,2^.

1. Securing Antarctica’s Environmental Future, University of Wollongong, Wollongong, NSW, 2522, Australia.
2. Centre for Sustainable Ecosystem Solutions, School of Earth, Atmospheric and Life Sciences, University of Wollongong, Wollongong, NSW, 2522, Australia.
3. Universidad de La Laguna, La Laguna, Canary Islands, Spain.

Corresponding author Sharon A Robinson [sharonr@uow.edu.au](mailto:sharonr@uow.edu.au)

# Supplementary methods

## Temperature and Microclimate data

Daily minimum and maximum air temperatures for Casey Station, Windmill Islands, East Antarctica were obtained from the Bureau of Meteorology (BOM) for Casey (station 300017, 1989–2022), with earlier observations from nearby Casey Tunnel (station 300006, 1969–1989). BOM data for the spring/summer season (1^st^ November to 31^st^ March) have been summarised in Fig. 2. Mosses are only active when free water becomes available, which occurs when snow and ice melt at temperatures above 0 °C. Based on this, days when maximum air temperatures exceed 0 °C for the spring/summer of each year were determined as an indicator of the length of each growing season. Shifts in the seasonal number of days above 0 °C were detected for years 1979 and 1993 using the cumulative sum control chart tool in JMP Pro v16.1 (SAS Institute Inc.). A shift in the number of days was indicated using decision limits set to three standard deviations in both positive and negative directions away from the mean.

Moss canopy temperatures were measured over a 16 day period in February 2022 at Antarctic Specially Protected Area No.135 (ASPA135) approximately 500 m east of Casey Station. The area encompasses rocky outcrops that are predominately ice-free over the austral summer, and melt streams from snow and ice support an area of lush moss turf in these ice-free areas, consisting of the endemic *Schistidium antarctici* and two cosmopolitan species, *Bryum pseudotriquetrum* and *Ceratodon purpureus.* The moss turfs undergo frequent freeze-thaw cycles that warp the moss layer to form complex micro-topographic structures, consisting of a series of ridges and valleys that are highly variable over scales less than 1 m (Lewis Smith 1999; Lovelock and Robinson 2002; Lucieer et al. 2014; Turner et al. 2014). The state of health of the mosses is also highly variable over these small scales and corresponds to the micro-topography, where stressed (red) or moribund (black) moss is more likely to occur on the micro-topographic ridges while the valleys tend to support healthy (green) moss (Lovelock and Robinson 2002; Robinson et al. 2018; Lucieer et al. 2014). Moss canopy temperatures were measured at 20 point locations at high temporal resolution (30-minute intervals) using five HOBO data loggers (Onset HOBO UX120-014M 4-Channel Thermocouple Logger) each connected to four thermocouple sensors (RS-Pro Type T IEC 0.315mm Thermocouple). Thermocouple sensors were inserted into the moss turf approximately 2 mm below the surface, within the photosynthetic canopy of the moss turf and pinned in place. The point locations for measuring moss canopy temperatures were selected to capture different micro-topographic positions within the moss turf and different states of moss health including healthy, stressed and moribund.

## Photoprotective pigments

Collection of samples and climate data is described in Dunn and Robinson (2006) for 1999-2000 and Turnbull and Robinson (2009) for 2002-2003. Photoprotective pigments and chlorophylls were extracted in acetone and quantified by High Performance Liquid chromatography (Shimadzu 10A VP series HPLC; Shimadzu Scientific Instruments, Australia). Following the initial extraction in 100% acetone, moss cell debris was re-extracted twice in 0.5 mL 80% acetone (20 minutes on ice in the dark) to ensure complete extraction of pigments (Dunn et al. 2004). The extract was made up to 2 or 3 mL with 100% acetone and filtered using a 0.45 µm PTFE syringe filter. Samples were stored at –20 °C for up to 48 h before analysis. Samples (20 - 100 µL) were injected at a flow rate of 2 mL min^-1^ and separated on a Spherisorb ODS1 column (Alltech, Sydney, Australia). Solvent A (acetonitrile: methanol: 0.1M TRIS HCl buffer, pH 8.0; 79:8:3) ran isocratically for 4 minutes followed by a 2.5 minute linear gradient to 100% solvent B (methanol: hexane; 4:1) which ran isocratically from 6 to 15 minutes. To maintain stable pressure the flow rate was reduced to 1.5 mL min^-1^ between 6 and 12 minutes and then ran at 1.5 mL min^-1^ until 15 minutes. The column was flushed with Solvent A between samples. Pigments were quantified by

integration of peak areas, detected at 440 nm using a photodiode array detector (Model SPD-M10AVP; Shimadzu), relative to pure chlorophyll (Sigma, Sydney, Australia) and carotenoid (Extrasynthase, Genay, France and VKI, Horsholm, Denmark) standards.

Total chlorophyll (Tchl: chlorophyll *a* + chlorophyll *b*) was reported on a fresh weight basis in the 2002-2003 season, while dry weight was used in 1999-2000. For this reason all pigment parameters shown here are expressed as ratios for consistency across both seasons: xanthophyll pool (VAZ/Tchl) and β-carotene (β-carotene/Tchl; mmols.mol^-1^) and the xanthophyll conversion state is expressed as the amount of Z relative to the VAZ pool (Z/VAZ).

Zeaxanthin/VAZ, β-carotene/Tchl and VAZ/Tchl were chosen as the most representative indicators of light acclimation and damage response in plants. The value for each of these three pigment indicators was plotted against collection date for each of the three moss species. A second order polynomial was fitted to the scatter plots to display seasonal trends (Fig. 7). Similarly, pigment ratios of three species across both summer seasons were plotted against water content, air temperature and UV index.

A MINI-PAM (Walz, Effeltrich, Germany) with leaf clip (model 2030B) Chlorophyll fluorescence was used to measure ɸPSII and ambient PAR directly adjacent to the sample. The effective quantum yield parameter (ɸPSII) was used to calculate electron transport rate (ETR) using the equation of Genty et.al (1989), which multiplies the efficiency of light use (ɸPSII) with the amount of light available to PSII. This was assumed to be 0.84 * 0.5, representing the amount of absorbed light and 0.5 for each photosystem (PSII and PSI).

ETR = ɸPSII $\times$ 0.5 $\times$ 0.84 $\times$ PAR

where PAR represents the photosynthetically active radiation in µmols photons.m.s⁻².

# Supplementary Figures

**
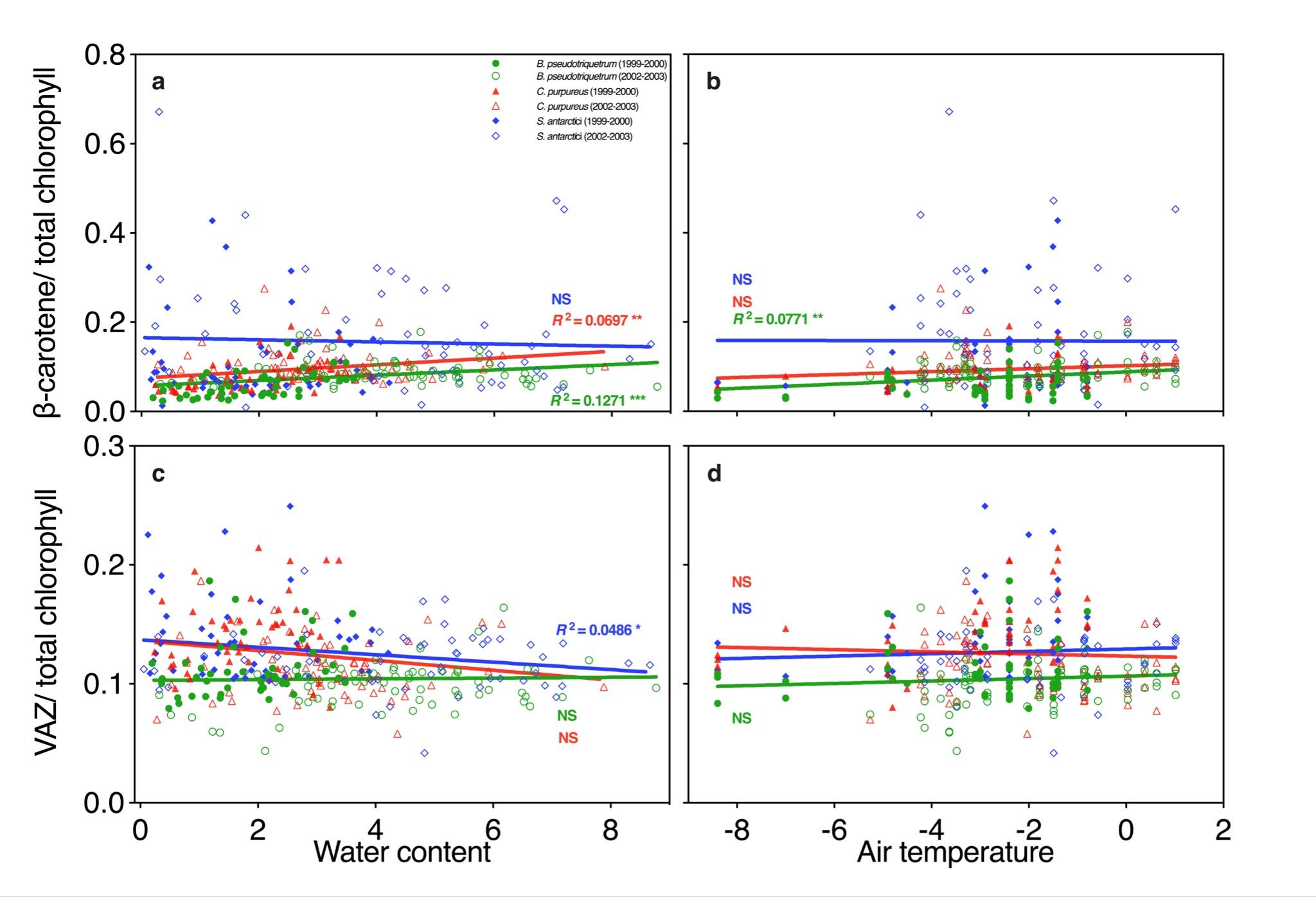
**

**Supplementary Figure 1** Relationship between photoprotective xanthophyll pigments β-carotene/chlorophyll and VAZ/chlorophyll in Antarctic mosses with **(a, c)**, moss water content (gH2O gdw^-1^) and **(b, d)** air temperature (°C). Associations for three moss species, *B. pseudotriquetrum* (*n* = 111, green)*,* *C. purpureus* (*n* = 110, red) and *S. antarctici* (*n* = 108, blue) measured across two seasons are shown.

**
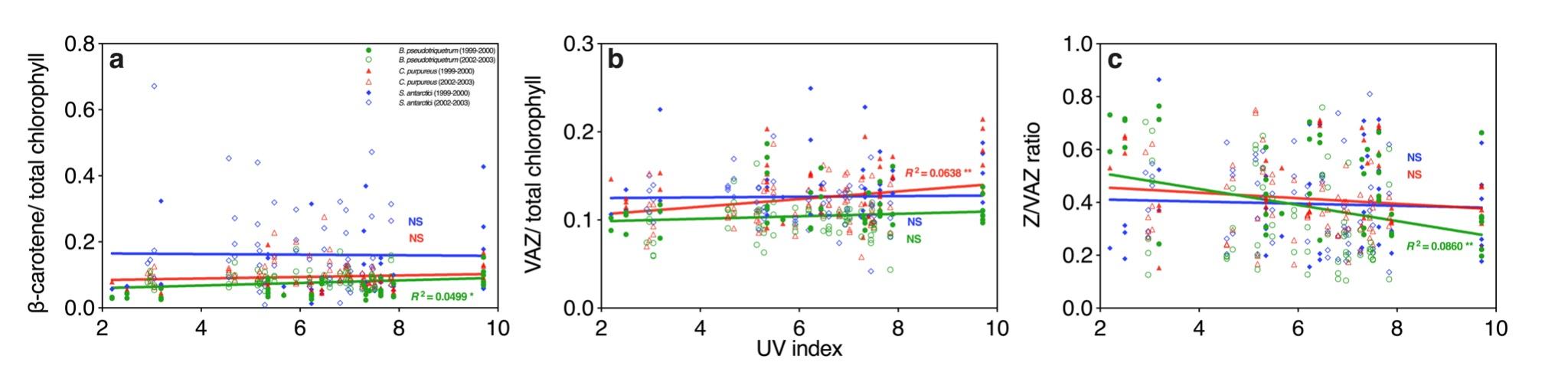
**

**Supplementary Figure 2** Relationship between photoprotective xanthophyll pigments and UV index **(a)** β-carotene/chlorophyll, **(b)**VAZ/chlorophyll and **(c)** Z/VAZ in Antarctic mosses. Associations for three moss species, *B. pseudotriquetrum* (*n* = 105, green)*,* *C. purpureus* (*n* = 104, red) and *S. antarctici* (*n* = 104, blue) measured across two seasons are shown.

**References**

Dunn J (2000) Seasonal variation in the pigment content of three species of Antarctic bryophytes. BSc. Honours, University of Wollongong, School of Biological Sciences

Dunn JL, Robinson SA (2006) Ultraviolet B screening potential is higher in two cosmopolitan moss species than in a co‐occurring Antarctic endemic moss: implications of continuing ozone depletion. Global Change Biol 12:2282-2296 <https://doi.org/10.1111/j.1365-2486.2006.01283.x>

Dunn JL, Turnbull JD, Robinson SA (2004) Comparison of solvent regimes for the extraction of photosynthetic pigments from leaves of higher plants. Funct Plant Biol 31:195-202 <https://doi.org/10.1071/FP03162>

Lewis Smith R (1999) Biological and environmental characteristics of three cosmopolitan mosses dominant in continental Antarctica. J Veg Sci 10:231-242 <https://doi.org/10.2307/3237144>

Lovelock CE, Robinson SA (2002) Surface reflectance properties of Antarctic moss and their relationship to plant species, pigment composition and photosynthetic function. Plant Cell Environ 25:1239-1250 <https://doi.org/10.1046/j.1365-3040.2002.00916.x>

Lucieer A, Turner D, King DH, Robinson SA (2014) Using an Unmanned Aerial Vehicle (UAV) to capture micro-topography of Antarctic moss beds. Int J Appl Earth Obs Geoinf 27:53-62 <https://doi.org/10.1016/j.jag.2013.05.011>

Robinson SA, King DH, Bramley-Alves J, Waterman MJ, Ashcroft MB, Wasley J, Turnbull JD, Miller RE, Ryan-Colton E, Benny T (2018) Rapid change in East Antarctic terrestrial vegetation in response to regional drying. Nat Clim Change 8:879-884 <https://doi.org/10.1038/s41558-018-0280-0>

Turnbull JD, Robinson SA (2009) Accumulation of DNA damage in Antarctic mosses: correlations with ultraviolet‐B radiation, temperature and turf water content vary among species. Global Change Biol 15:319-329 <https://doi.org/10.1111/j.1365-2486.2008.01739.x>

Turner D, Lucieer A, Malenovský Z, King DH, Robinson SA (2014) Spatial co-registration of ultra-high resolution visible, multispectral and thermal images acquired with a micro-UAV over Antarctic moss beds. Remote Sens 6:4003-4024 <https://doi.org/10.3390/rs6054003>
